# Can there be overly meaningful lives? Conflicts between meaning in life and other values

**DOI:** 10.3389/fpsyg.2022.946648

**Published:** 2022-10-13

**Authors:** Iddo Landau

**Affiliations:** Department of Philosophy, University of Haifa, Haifa, Israel

**Keywords:** eudaimonic wellbeing, meaning in life, philosophy, subjective happiness, value

## Abstract

This is a philosophical paper that heeds psychological work on meaning in life, and hopes to acquaint both psychologists and philosophers more with each other’s work and enhance a dialogue between them. Many works on meaning in life in philosophy and in psychology have already focused on the relations between meaning in life and specific values such as happiness (subjective wellbeing), authenticity, morality, knowledge, and artistic creation. This paper discusses the general structure of the relation between both objective and subjective meaning in life and other values, and emphasizes ways in which such values sometimes conflict with rather than enhance objective or subjective meaning in life. The paper argues that, because of such conflicts, there are cases in which we should refrain from augmenting the objective or subjective meaning in our lives and even seek to decrease it; there can be overly meaningful lives. The paper concludes with some practical implications of this discussion.

## Introduction

This paper is written by a philosopher, not a psychologist. One of the paper’s central aims, following Baumeister’s (2022, p. 426) observation that “philosophy can learn from psychology … psychology can benefit much from continued input from philosophers,” is to encourage both philosophers and psychologists working on meaning in life to become more acquainted with each other’s work. I agree with Baumeister that researchers of meaning in life in both disciplines can learn much from familiarizing themselves with the work done in what might be called “the other discipline.” Much of it may be new and perhaps seem odd, but it may bring to light and challenge long-held implicit presuppositions, suggest new paradigms, and introduce ideas for further research. This, then, is a philosophical paper that aims to heed psychological work on meaning in life, and hopes to acquaint both psychologists and philosophers more with each other’s work and enhance a dialogue between them.

I should distinguish, however, the aim of the present paper from that of an important and interesting effort to create a new interdisciplinary field that would interweave psychological empirical measured evidence with philosophical assumptions and theories. For example, Cokelet and Fowers (2019) discuss, among other issues, the possibility of developing models that would respond to questions about the normative significance of virtues. Fowers et al. (2021) examine how positive psychology and personality psychology relate to virtue research in a way that treats virtue as empirically verifiable and measurable. Layder (2021) mentions, among other issues, the need to reject the distinction between theory and method, as well as to develop research strategies that link global properties of social reality with local properties of research data. Prinzing (2021) argues that much in positive psychology is implicitly value-laden anyway, and suggests that these values should be explicitly endorsed. It seems to me that, at present, it is too early to know whether this interdisciplinary subfield will indeed emerge. If it does, the discussion in this paper may prove to be relevant to it. However, for now, the discussion in this paper treats psychology of meaning in life and philosophy of meaning in life as distinct fields, and suggests that, as such, both philosophers and psychologists may find their thoughts and research enriched by learning of each other’s work.

One general difference between much of the contemporary psychological and philosophical discussions of meaning in life (henceforth just “meaning”) is that psychological discussions on meaning largely focus on *sensed* or *conceived* meaning, which philosophers often call “subjective meaning.” Philosophical discussions, too, focus on subjective meaning, but also often on what they take to be objective meaning, which is the meaning that aspects of life are taken to have unrelated to their being sensed or conceived as meaningful. To clarify the distinction consider the example of Ignaz Semmelweis’s life (Benatar, 2022, p. 435). Semmelweis insisted, against the accepted medical and scientific views of his time in the 1840s, that obstetricians should wash their hands between surgeries. His insistence, which pioneered modern antiseptic procedures, led to millions of lives being saved but also to his professional ostracization, a nervous breakdown, and forced hospitalization in a mental asylum. Many philosophers would hold that Semmelweis had an objectively meaningful life even if, historically or in a thought experiment, he experienced his life as meaningless. Semmelweis, or a version of him, may have had a subjectively meaningless but objectively meaningful life. Likewise, consider the example of a bored or depressed Mother Teresa (following Metz, 2013, p. 135). The historical Mother Teresa was, in fact, occasionally depressed or deeply anxious, feeling that she was not “in the grace of God” and describing herself as feeling lost and in darkness (Teresa and Kolodiejchuk, 2007). Perhaps she did not sense her life as meaningful at those times, but many philosophers of meaning in life would still take her life to have been objectively meaningful even at those times because of her impressive moral achievements and the many lives she saved. Even if all that were not true of the historical Mother Teresa, we can, as a thought experiment, think of someone else, a “version” of Mother Teresa who succeeded in saving a million lives while not taking her life to be meaningful (perhaps she just acted out of duty). Many philosophers of meaning in life would consider such a person to have had a subjectively meaningless but objectively meaningful life.

Just as there can be subjectively meaningless but objectively meaningful lives, so there can be subjectively meaningful but objectively meaningless lives. An example of the latter might be the life of a guru-worshiping cult member who loses his autonomy and critical thinking but gains a sharp sense of meaning when working hard to expand his dishonest guru’s Rolls Royce collection. Likewise, consider a young person who joins the SS, experiencing a sharp sense of increased meaning while having his autonomy and critical thinking diminished in the military frameworks he is part of, fighting for a worthless, hideous cause, and committing terrible crimes. He, too, may have a subjectively meaningful and objectively meaningless life. This paper discusses both objective and subjective meaning.

While considering psychological and philosophical work, the paper elaborates on conflicts that can arise in various circumstances between objective and subjective meaning and other values or aspects of life, and thus on how lives can at times be overly objectively and subjectively meaningful. We want our lives to be meaningful, but that is not the only thing we want from them. We also want them to be, among other things, autonomous, authentic, interesting, happy, and moral, and to include love, knowledge, friendship, aesthetic experiences, and many other positive values. Several philosophical works have already focused on the relations between objective and/or subjective (henceforth just “objective or subjective”) meaning and a specific value such as morality (Thomas, 2005), happiness (Metz, 2009), creativity (Matheson, 2016), love (Kronqvist, 2017), wonder (Schinkel, 2019), forgiveness (Allais, 2022), or gratitude (Manela, 2022). Likewise, several psychological works have already focused on the relation between (mostly subjective) meaning and a specific value such as happiness (subjective wellbeing) (Baumeister et al., 2013), creativity (Kaufman, 2018), optimism (Yu and Chang, 2019), gratitude (Kleiman et al., 2013), self-compassion (Suh and Chong, 2022), empathy (Komisar and McFarland, 2017), or authenticity (Lutz et al., 2022). The aim of this paper, however, is to discuss the general structure of the relation between objective or subjective meaning and other values, underscoring the ways in which other values and objective or subjective meaning sometimes conflict with and diminish rather than enhance each other. Further, the paper argues that, because of these conflicts, there are cases in which we should refrain from trying to augment objective or subjective meaning in our lives and even seek to decrease them—there can be overly meaningful lives. Thus, this paper focuses on the more problematic side of meaning in life, which both psychological and philosophical research hardly discuss. The paper suggests that, although often helpful and positive, in some circumstances meaning can be harmful and problematic.

## Conflicts between objective meaning and other values

In contemporary philosophical analyses of objective meaning, the most accepted view by far is that value primarily constitutes life’s meaning (e.g., Hepburn, 2000, p. 262; Joske, 2000, pp. 287–290; Cottingham, 2003, p. 31; Brogaard and Smith, 2005, pp. 443–444; Wolf, 2010, pp. 13–33; Kauppinen, 2012, pp. 353–356, 361–367; Metz, 2013, pp. 220–239; Landau, 2017, pp. 6–16; for dissenting views, see Goldman, 2018, pp. 116–151; Repp, 2018; Seachris, 2019; Thomas, 2019; for replies defending the value view, see Metz, 2019, pp. 409–411; Landau, 2021). To make life meaningful, we try to increase the overall value in different spheres of life. These may include, for example, some or all of the following: aesthetic enjoyment, wisdom, morality, love, subjective happiness, and social recognition. When these or other spheres of value together show a sufficiently high degree of overall value, we take life to be meaningful. If they do not together pass some threshold of overall value, we do not take the life in which they appear to be meaningful. Different people have different views about which spheres of our lives are of value. For example, some may think that scholarship is but financial success is not a sphere of value. Others may take the opposite view or see both as spheres of value but one of them as more valuable and, thus, contributing more to life’s meaning than the other. I will not discuss the specifics of these issues here; for the purposes of the present analysis, it is sufficient that the general structure of the relation between meaning and other values be accepted.

Note, however, that a life can have overall value sufficient to make it objectively meaningful even if many values appear in it only to a low degree or not at all, as long as some of the values that do appear in it are of a sufficiently high degree. For example, while we take music, literature, the visual arts, love, friendship, and knowledge to enhance objective meaning, we can also think of highly meaningful lives that incorporate very few or even one of these values. For instance, we would consider the life of Mother Teresa to have been objectively meaningful thanks to her moral achievements even if some or all of the other values mentioned above had been absent from her life or appeared just to a minimal degree. Similarly, we would consider Mozart’s life to have been objectively meaningful thanks to his contribution to music even if he were friendless, loveless, showed no interest in other arts, and had no substantial knowledge in other fields such as science of mathematics.

Further, enhancing a certain value in a life will not always enhance the overall objective meaning in that life. Take, for example, Autonomy. Autonomy is a positive value, but there are cases in which people’s autonomous choices decrease rather than increase their lives’ objective meaning. Consider, for instance, a person who, upon gaining a higher sense of personal autonomy, or gaining more practical autonomy in her life, starts embezzling money from her clients and abusing her family, thus diminishing her life’s objective meaning. Similarly, there are people whose lives become more objectively meaningful because their autonomy is diminished, that is, because they are forced to do certain things (as long as their degree of autonomy does not fall to a robot-like level). Consider a person who was drafted against his will to fight in World War II and because of this interacted with some deep and thoughtful people who taught him much, and moreover took part in a pivotal campaign and saved many lives, all of which considerably increased the objective meaning of his life. Had he not been drafted, all this would not have happened and he would have had a less objectively meaningful life. In some circumstances, then, autonomy and objective meaning are *competing* values and we have to choose whether we would prefer to diminish objective meaning and enhance autonomy or to diminish autonomy and enhance objective meaning.

Likewise, consider happiness (or subjective wellbeing), that is, the psychological state of contentment, lightness of heart, and cheerfulness. As a positive value, it seems that the more subjective happiness is present in a life, the more that life is objectively meaningful. Nevertheless, objective meaning and happiness do not always come together; people can lead unhappy but objectively meaningful lives. Take, for example, Søren Kierkegaard. His life seems to have been objectively meaningful: he published many important philosophical works, had an immense positive effect on both existentialist and religious thought and, through them, on the lives of many people, and proved to be probably one of the 20 most important Western philosophers who ever lived. Thus, he seems to have as good a claim as any to having led a very objectively meaningful life. However, according to his biographers, he led an unhappy, even tortured life (Hannay, 2003; Garff and Kirmmse, 2007). It is likely, of course, that he did experience some contentment because of the meaningful aspects of his life. But all in all, his life was not a happy one; he had a meaningful but unhappy life. Moreover, it seems that some of the qualities that made him unhappy also contributed to his accomplishments and, thus, to his life’s meaning. Kierkegaard’s extreme, perfectionist expectations of himself, relentless honesty with himself and others, and uncompromising nature arguably made him unhappy, lonely, and tortured, on the one hand, but also a great, original philosopher on the other hand. It may well be that had he been less of a perfectionist, etc., his life would have been happier but less meaningful. The same seems to be true of Emily Dickinson. For the majority of her adult life, until her death at age 56, she lived in self-imposed reclusiveness. Her poetry—many of her almost 1800 poems discussing death or unfulfilled love—suggests that in many aspects of her life she was an unhappy person, something confirmed by her biographers (Sewall, 1998; Kirk, 2006). Yet, she is considered with good reason to be a unique and important poet and, thanks to that, to have had an objectively meaningful life. It seems that some of the emotional forces that led to her isolation and unhappiness, such as her extreme sensitivity, intensity, and uneasiness with people, also led her to write her exceptional poetry. Thus, although there are many circumstances in which subjective happiness and objective meaning do not compete but enhance each other, there are also circumstances in which increasing one decreases the other.

Similar relations exist between objective meaning and other values, such as authenticity, morality, truth, camaraderie, health, longevity, and love. All these values in many circumstances enhance life’s objective meaning, yet in other circumstances conflict with it. We can think of circumstances in which, say, one’s inauthentic appreciation of her supervisor’s dull jokes could result in receiving an academic fellowship and, thus, an excellent education that would enable her to considerably increase objective meaning in her life. Somewhat similar examples, but with immoral behaviors (such as telling a small lie, committing a small theft, or failing to keep a promise), that allow one to receive an excellent education show that failures in moral behavior may sometimes enhance objective meaning, while holding to one’s moral standards may in some circumstances decrease it. Objective meaning may in some circumstances also conflict with camaraderie: pursuing one’s own interests or developing one’s gifts to their maximum sometimes requires relative isolation and individual action that sets one apart from others. Likewise, when one sacrifices one’s health or life for a noble cause, objective meaning competes with health and longevity. Mahatma Gandhi, Martin Luther King, Jr., Anwar Sadat, and Yitzhak Rabin are examples of people who would have had longer lives if they had not acted in ways that improved the world and rendered their lives more objectively meaningful. What has been shown in these examples is true also of the relation between objective meaning and other values; I have not so far succeeded in finding any value that does not in some circumstances enhance objective meaning yet in others decrease it.

But cases in which enhancing some values in life diminishes its objective meaning seem to conflict with what was explained above about the nature of life’s objective meaning. If life’s objective meaning is based on value, should we not expect the enhancement of value always to increase objective meaning? The reply is that values also affect each other, and when they diminish other values, they can also diminish objective meaning. Take, for example, love: suppose that one devotes time and energy to one’s love life, so that there is now more love in one’s life. Enhancing love may well enhance objective meaning, as there is now more value in one’s life. Enhancing love may also enhance other spheres of value that enhance life’s objective meaning: the love in one’s life may increase optimism, goodwill, and energy, enabling one, say, to study better, appreciate natural beauty more deeply, and behave more morally toward others. However, in some circumstances, progress in the sphere of love may *decrease* the value in other spheres of value and, through them, the overall objective meaning of one’s life. For example, one may, while focusing on love, disregard one’s studies and discontinue one’s supererogatory moral contributions. While the value in some spheres increases (and contributes positively to objective meaning), the value in other spheres may decrease to such an extent that the *overall* value, and therefore the objective meaning in one’s life, diminishes. Of course, all other things remaining equal, increase in any value enhances objective meaning. But in almost all cases, all other things do *not* remain equal; they are affected in many ways, some of them positive, others negative. Thus, increase in a value can lead to a decrease in objective meaning and vice versa.^1^

## Conflicts between subjective meaning and other values

Psychological research on what philosophers call subjective meaning suggests that it, too, can conflict with many values. Consider, first, happiness (or subjective wellbeing, understood as having more pleasant than unpleasant emotional states as well as a general positive assessment of one’s life as being more emotionally pleasant than unpleasant). Baumeister et al. (2013) have shown that although subjective meaning and happiness are positively correlated, they are distinct, have many different predictors, and do not always coincide. For example, higher anxiety, stress, and worry associate with lower happiness but with higher subjective meaning. Thinking about the future (and, more generally, integrating past, present, and future) also link to higher subjective meaning but to lower happiness. Baumeister et al. (2013) also show that subjective meaning has more to do with being a giver rather than a taker whereas subjective happiness has more to do with being a taker rather than a giver. Expressing one’s self relates to subjective meaning but hardly to happiness. In three percent of the reported cases subjective meaning was due to bad events that happened to participants in the survey (Baumeister et al., 2013, p. 515). Thus, notwisthstanding the considerable overlap between happiness and subjective meaning, enhancing one of them may decrease the other.

What Baumeister et al. (2013) found of the relation between subjective meaning, on the one hand, and happiness, worry, anxiety, tension, and bad events in life, on the other hand, also holds for the relation between subjective meaning and other values. In some cases, traumas lead to posttraumatic growth that enhances meaning (Cann et al., 2010; Triplett et al., 2012). Hatred toward other groups and institutions can stimulate subjective meaning (Elnakouri et al., 2022). Authoritarian worldviews predict meaning (Womick et al., 2019). King and Hicks (2021, p. 572) hypothesize that “one reason even maladaptive worldviews may be difficult to change is that they imbue life with meaning.” We see, then, that in some cases higher subjective meaning conflicts with positive values and instead relates to negative ones.

This should not be surprising. Think, again, of the example of the guru-worshiper or the SS soldier whose lives show high subjective meaning but, relatedly, many negative values. Likewise, although the examples presented in the previous section focused on objective meaning, they also hold, with small changes, for subjective meaning. Kierkegaard or Dickinson (or people similar to them) may well have also strongly experienced subjectively meaningful lives through their intense (and possibly obsessive) commitment to their philosophy and poetry that cost them so much else in their lives. The examples in the previous section of the possible conflicts between objective meaning and authenticity, morality, camaraderie, and other values could also show, with small changes, how subjective meaning can conflict with many positive values and relate to negative ones.

It is easy to explain how this can happen. Most contemporary psychological discussions of subjective meaning emphasize purposefulness, significance/mattering, and coherence/comprehension as constituting subjective meaning (e.g., Steger et al., 2006; Heintzelman and King, 2014; George and Park, 2016; Martela and Steger, 2016). Purposefulness can coexist with and even can be enhanced by some degrees and types of negative values such as worry, anxiety, tension, pain, bitterness, hate, radical acceptance of authority, loneliness, or single-mindedness. For example, a person who is more worried, anxious, and single-minded may become more purposeful in her efforts to advance some good, or some bad (e.g., racist), political agenda. In some circumstances, purposefulness may also conflict with some positive values such as serenity, love, self-acceptance, happiness, spontaneity, or compassion. Negative values such as worry, anxiety, tension, pain, bitterness, hate, radical acceptance of authority, loneliness, or single-mindedness can similarly enhance behaviors that are impactful, that “matter,” or are “of significance” to one’s human and physical environment, and thus also contribute to one’s sesnsing one’s life as “mattering.” Likewise, once the guru worshipper or the SS volunteer subdue their tendencies toward critical rationality, intellectual and emotional openness, and tolerance, accepting instead authoritarianism and blind commitment, they may sense their lives and the world around them as more coherent and comprehensible than before. In fact, critical thinking, openness, tolerance, and rejection of authoritarianism are likely to lead to more complex, nuanced, and uncertain worldviews, which may diminish one’s ability to experience life and the world as simple and, as such, easily comprehensible and coherent. All this has to do with the fact that purposefulness, significance, and coherence are largely value neutral. They relate to both positive and negative values. Hence, in some cases, enhancing subjective meaning will come at the price of diminishing some other important values in life, and vice versa.

## Conflicts between objective or subjective meaning and eudaimonic wellbeing

I have discussed up to now possible conflicts between objective or subjective meaning and a variety of values. But in recent years, a new important notion, *eudaimonic wellbeing*, has emerged in the psychological literature, and it might be thought that things are different with it. Eudaimonic wellbeing is distinguished from hedonic wellbeing: while the latter has to do with a high ratio of positive to negative affect and a high rate of satisfaction with one’s life, the former has to do with what is conceived of as living well, realizing one’s virtuous potential and fulfilling one’s true nature (Deci and Ryan, 2008, pp. 1-2).^2^ There are different conceptualizations of eudaimonic wellbeing. I will discuss here three of them, and argue that they, too, can conflict with objective or subjective meaning, so that increasing objective or subjective meaning may decrease eudaimonic wellbeing, and increasing eudaimonic wellbeing may decrease objective or subjective meaning.

Ryan et al. (2008) typify eudaimonic wellbeing as having to do with engaging in intrinsic rather than extrinsic goals (e.g., health, personal growth, community, and relationships rather than power, wealth, fame, and image), acting in autonomous and volitional rather than in controlled ways, and acting in ways that satisfy the psychological needs for competence, relatedness, and autonomy. But some of these qualities may well be in conflict with objective meaning. We would probably consider Shakespeare’s life to be of high objective meaning even if we found that, to a significant degree, he wrote for the sake of extrinsic goals such as wealth or fame. It may well be that increased interest in extrinsic goals such as wealth and fame, which would have diminished eudaimonic wellbeing, would have led him to create even more or better work, while diminished interest in wealth or fame, which would have enhanced his eudaimonic wellbeing, would have led him to a lower volume and poorer quality of work. His interest in art for art’s sake, or perhaps in personal growth, as intrinsic goals might have been low and insufficient to motivate him to achieve what he did.

We would also take Shakespeare’s life to be objectively meaningful even if we learned that he did not create wholly autonomously or volitionally but partly compulsively, perhaps feeling that he just had to write or that a muse was creating through him. Diminishing his possible compulsiveness and increasing his volitional and autonomous behavior might have enhanced his eudaimonic wellbeing but decreased meaning (since he might have chosen to focus less on his art and thus create less), while increasing his compulsiveness might have diminished his eudaimonic wellbeing but made his life more objectively meaningful. Likewise, we would continue to see Shakespeare’s life as objectivley meaningful even if we discovered that he was a loner who did not act in ways that satisfied his psychological need for relatedness. Again, in some cases and circumstances, encouraging him to stop being a loner and to satisfy his psychological need for relatedness could have increased his eudaimonic wellbeing but decreased objective meaning in his life (since he would have spent more time with friends and less on artistic creation).

In various circumstances, the version of eudaimonic wellbeing Ryan et al. (2008) present will also be in conflict with subjective meaning. The guru-worshiper or the SS soldier may have such a strong sensation of meaning in their lives because they forgo their autonomy and volitional behaviors by succumbing completely to and identifying with some leader’s will, thus acting in controlled and heteronomous ways. Had they been more autonomous in thought and in deed, they might have not been so focused on the goal set by their leaders, the world might have made less sense to them, and they might have had less impact on their environment (or “mattered” less), and also sensed their lives as less purposeful, comprehensible, and “mattering.” Thus, their lives would have been of higher eudaimonic wellbeing but of lower subjective meaning. Again, in some cases, we would have to choose between enhancing objective or subjective meaning, on the one hand, and enhancing eudaimonic wellbeing, on the other. Enhancing one of them will decrease the other.

Ryff and Singer (2008, pp. 20–23) discuss eudaimonic wellbeing as consisting of autonomy, self-acceptance, positive relations with others, personal growth, purpose in life, and environmental mastery. As just shown when discussing Ryan et al. (2008), autonomy (with eudaimonic wellbeing) can decrease while objective or subjective meaning increases and vice versa. And the examples of Kierkegaard and Dickinson (or versions of them) suggest that it is possible to have lives in which self-acceptance and positive relations with others decrease (and, thus, eudaimonic wellbeing decreases) while objective or subjective meaning increases, and vice versa. Thus, again, for some people, in some circumstances, higher meaning would come at the price of lower eudaimonic wellbeing, and higher eudaimonic wellbeing at the price of lower meaning.

Waterman et al. (2008) relate eudaimonic wellbeing to activities that have to do with self-determination, activities that lead agents to feel fulfilled, and activities that are expressive of who their agents really are. The notion of self-determination that Waterman and colleagues employ seems quite similar to that of autonomy discussed above. The examples of Kierkegaard and Dickinson (or versions of them) suggest that it is possible have a life of high objective or subjective meaning while feeling quite unfulfilled. Feeling unfulfilled may even motivate one to achieve and excel (thus gaining higher objective meaning) in the hope that this may finally lead to experiencing fulfillment. Feeling unfulfilled may also direct one to focus on purpose, “mattering,” and comprehension, in the hope that experiencing high subjective meaning may compensate for the lack of a sense of fulfillment. Likewise, the SS soldier and the guru-worshiper may well be acting in ways that are expressive of who they really are. The former may have a murderous, sadist, highly aggressive personality, and the latter an obedient, gullible, and eager to please personality. Thus, their acting in ways that enhance their eudaimonic wellbeing may diminish objective meaning in their lives, and vice versa. Again, eudaimonic wellbeing can conflict with both objective and subjective meaning.

## Conflicts between objective and subjective meaning

Up to now, I have discussed ways in which objective or subjective meaning can conflict with *other* values. But it is worth noting that in some circumstances objective and subjective meaning also conflict with each other, so that increasing one of them decreases the other. Subjective meaning can enhance objective meaning since it can be seen as part of what makes life objectively meaningful (Metz, 2013, pp. 183–184), and the effort to maintain subjective meaning can lead people to behave in objectively meaningful ways. But subjective meaning can also diminish objective meaning. As the examples of the guru-worshiper and the SS soldier show, in order to maintain strong subjective meaning people sometimes behave in ways that render their lives *less* objectively meaningful. Sought or experienced subjective meaning can easily lead people astray. We would also see the life of the SS soldier as less objectively meaningful if he experienced his actions as meaningful than if he experienced his actions as non-meaningful. And just as subjective meaning in some cases diminishes objective meaning, so objective meaning can in some cases diminish subjective meaning. Consider a person who performs a heroic act against a tyrant and is consequently put for a long time in solitary confinement that leads her to experience her life as meaningless.

## Deciding between meaning and other values

When meaning and other values conflict, we have to make choices. Much in these decisions depends on the specific circumstances and on the particular degrees of meaning and of the other values at stake. Let us take first, again, objective meaning. Consider the cases of Kierkegaard and Dickinson. Given a choice between educating a child either into a Kierkegaard or into a person (call him Kierkegaard*) whose life is much subjectively happier yet somewhat less objectively meaningful than Kierkegaard’s, most would likely prefer the latter option. Perhaps, if the choice were between a Kierkegaard and a happy but very stupid and vulgar Kierkegaard*, we would prefer Kierkegaard. But we would not prefer Kierkegaard if the alternative were a plausibly intelligent and cultured Kierkegaard* who has a much happier but less objectively meaningful life than Kierkegaard’s. Some may have different intuitions and still prefer Kierkegaard’s life to that of Kierkegaard*. But they, too, could fill in the specific details such that they would prefer a particular version of Kierkegaard* to Kierkegaard. By decreasing the difference in life’s objective meaning between Kierkegaard* and Kierkegaard, so that Kierkegaard*’s life is almost as objectively meaningful as Kierkegaard’s, and by increasing the difference in happiness, so that Kierkegaard*’s life is much happier than Kierkegaard’s, there would be a point at which probably almost all would opt for increasing happiness even if it entailed decreasing objective meaning. The same, I believe, is true of Dickinson. Most, I believe, would prefer to educate their daughter to be less tortured, intense, and unhappy than Dickinson was, even if that meant that she would end up with a somewhat less objectively meaningful life. We will not always opt for objective meaning rather than happiness. The same is true of objective meaning vs. authenticity, longevity, love, and all the other values mentioned above, including eudaimonic wellbeing.

I suggest that the same holds for subjective meaning. It, too, is important and valuable, but when in conflict with other values, it does not override, at any degree, any other degree of other values (or their combination). Consider, again, the cases of Kierkegaard and Dickinson, now focusing only on the subjective meaning they may have experienced. I believe that most, again, would prefer not to raise a child to be as lonely, tense, and upset as Kierkegaard and Dickinson were, even if we knew that this would allow them to experience their lives as highly subjectively meaningful. As with objective meaning above, we could play with the degrees of subjective meaning and of the other values. Likewise, we would not want to raise a child who would be a guru-worshiping fanatic or a *Führer*-dedicated SS soldier even if that would allow them very strong, prolonged, experiences of meaning. Subjective meaning is important and valuable. But it is not always, and at any degree, more important than other values, including those that constitute eudaimonic wellbeing, in any degree or combination.

But how, in cases of conflict, do we choose between (or find the optimal balance of) objective or subjective meaning and other values? First, we tend to be willing to “pay” with a small decrease in one value if this leads to a significant increase in another value. For example, we would tend to agree to a small decrease in objective or subjective meaning if that would allow us a very large increase in happiness, and vice versa. Consider a person who volunteers in a foreign country for an important philanthropic cause. She does not enjoy her volunteer work because she is overly stressed and homesick. But she knows that her special skill is desperately needed and produces much good so that her volunteer work renders her life both objectively and subjectively meaningful to a high degree. If the degree of attained objective or subjective meaning had been lower or the degree of unhappiness higher, she might have not agreed to “pay” with happiness for the gained objective or subjective meaning as she does. Likewise, a person who considers whether to blow the whistle on his superiors, thus behaving morally but risking decline in the degree of the overall objective or subjective meaning in his life (because of the risk to his employment, peace of mind, relationships, and his ability to focus on his poetry), may judge that some degree of moral improvement is not worth the price in meaning while some other is.

Second, much depends on the estimation of the likelihood of success in the endeavor. The person who pays now with inauthenticity to achieve objective or subjective meaning in the future has to estimate how probable it is that, thanks to her pretended enjoyment of the supervisor’s dull jokes, she will indeed receive the education she longs for. Likewise, a person who forgoes some of what he considers meaningful in his life in order to develop a love affair has to estimate how probable it is that the love will indeed develop and subsist.

Third, we work by thresholds. There is the threshold between what we take to be sufficient and insufficient objective and subjective meaning, as well as the threshold between what we take to be sufficient and insufficient happiness, morality, autonomy, and authenticity. When we feel quite happy, we may be ready to sacrifice more happiness than when we feel that we are on the verge of becoming unhappy; in other words, we may be more reluctant to sacrifice even a small amount of happiness if we think that doing so might result in falling below our threshold of happiness into what we consider unhappiness. The same would be true if we were slightly below rather than slightly above the threshold of happiness. We may feel that if we do not prefer happiness to objective or subjective meaning in such circumstances we will endanger our chances of crossing back over the threshold and recovering our happiness. However, if we are farther down the scale of happiness, we may so despair of attaining happiness that we do not care about it much anymore and would be willing to give up even more happiness to attain more meaning.

In cases of conflict between meaning and other values, then, we should not always opt for meaning. In some cases, there are good reasons to prefer meaning, and in others, there are good reasons to prefer other values. This means that, contrary to common wisdom, we should not always try to maximize objective or subjectve meaning in our lives. In some circumstances, moreover, we should even try to decrease meaning.

## Practical implications

The discussion presented in this paper also has some practical implications. First, since objective and subjective meaning have many possible sources, each of which can by itself make a life objectively or subjectively meaningful, it would serve one well to keep in mind that when some or many sources of meaning cease to be available, a sufficiently high degree of even one or two of the others can still maintain life as meaningful. This is an important point to remember for those who believe that their lives have become meaningless because sources of meaning that they have become habituated to, such as their career, artistic activity, love, or social activism, have ceased to be helpful or are no longer available. Although the shift to other sources of meaning can be difficult and unintuitive at first, it is important to remember that, after some time, these other sources for life’s meaning may make life as, or even more, meaningful.

Second, it is important to distinguish well between subjective and objective meaning; they are distinct, and the presence or absence of one of them does not entail the presence or absence of the other. Thus, people who sense their lives as meaningless should note that their problem need not have to do with objective meaninglessness. As in the example of the depressed Semmelweis, it might be limited only to the subjective sphere. Likewise, those who sense their lives as highly meaningful will do well to critically examine whether their lives indeed are also objectively meaningful (as in the example of the guru worshipper). Strong sensations of meaning or of meaninglessness (as strong sensations and feelings in general) may decrease the tendency to think clearly and critically, thus lowering the ability to conceive correctly the degree to which life is also objectively meaningful or meaningless.

Third, the value in objective and subjective meaning notwithstanding, since they interrelate with other values both positively and negatively, those who want to increase objective or subjective meaning in their lives should examine whether they do not thereby too greatly diminish other values important to them (including their happiness or eudaimonic wellbeing). In objective and subjective meaning, too, more is not always better. We should try to strike a balance between objective or subjective meaning and other values that are important to us. This means that, in order not to diminish too strongly other values, in some circumstances we should refrain from enhancing objective or subjective meaning beyond a certain degree, and in others even diminish them if this is necessary to allow us to enjoy some other values. We should acknowledge that there can be overly meaningful lives.

## Ethics statement

Ethical review and approval was not required for the study on human participants in accordance with the local legislation and institutional requirements. Written informed consent for participation was not required for this study in accordance with the national legislation and the institutional requirements.

## Author contributions

The author confirms being the sole contributor of this work and has approved it for publication.

## Footnotes

1 I have discussed here objective meaning in life as based on value in general, but what has been said here also holds, mutatis mutandis, for other and more specific views of objective meaning as based on value, such as Metz’s (2013, pp. 219–248) fundamentality theory as well as Wolf’s (2010) and Kauppinen’s (2012) views.

2 Note, however, that unless it is assumed that one’s true nature is virtuous, fulfilling one’s true nature may conflict with realizing one’s virtuous potential.
